# A patient with post-Fontan operation underwent left hepatectomy and caudate lobectomy for hepatocellular carcinoma: a case report

**DOI:** 10.1186/s40792-020-00866-1

**Published:** 2020-05-19

**Authors:** Satoshi Nemoto, Shun-ichi Ariizumi, Yoshihito Kotera, Akiko Omori, Shingo Yamashita, Taka-aki Kato, Hiroto Egawa, Masakazu Yamamoto

**Affiliations:** Department of Gastroenterological Surgery, Tokyo Women’s Medical University, 8-1 Kawada-cho, Shinjuku-ku, Tokyo, 162-8666 Japan

**Keywords:** Fontan-associated congestive liver cirrhosis, Hepatocellular carcinoma, Central venous pressure, Surgical resection, Reverse Trendelenburg position

## Abstract

**Background:**

The Fontan procedure has been widely accepted for children with single ventricle physiology and guarantees survival rates of approximately 80% at age 20 years. However, there have been cases of Fontan-associated liver disease (FALD) caused due to congestion, along with recent reports of the development of hepatocellular carcinoma (HCC) in younger patients with FALD. The literature consists of only five previous case reports of patients who underwent hepatectomy for HCC due to poorer cardiac function and liver cirrhosis caused due to congestion.

**Case presentation:**

The patient was a 37-year-old woman who presented with epigastralgia. Computed tomography (CT) revealed a liver tumor, 8 cm in diameter, in the caudate lobe. Liver damage was A, with an indocyanine green retention rate of 6% at 15 min. The levels of alpha-fetoprotein (AFP) and protein induced by vitamin K antagonists-II (PIVKA-II) were elevated to 81,663 ng/ml (normal < 10 ng/ml) and 238 mAU/ml (normal < 40 mAU/ml), respectively. Left ventricular ejection fraction was 56%, and central venous pressure (CVP) was 12 mmHg. Left hepatectomy and caudate lobe resection were successfully performed in the reverse Trendelenburg position which reduced the CVP. The total operation duration was 450 min, with a total blood loss of 3200 ml. The patient’s postoperative course was uneventful, and she is still alive 16 months after surgery.

**Conclusions:**

First left hepatectomy with caudate lobectomy during reverse Trendelenburg position which reduced the CVP was performed in a patient with HCC and FALD.

## Background

The Fontan procedure has been widely accepted for children with single ventricle physiology [1]. However, it has also been reported that Fontan-associated liver disease (FALD) develops due to congestion with the growth of these children who survive for a long time after the Fontan procedure [2]. In addition, development of hepatocellular carcinoma (HCC) in young patients with FALD has been recently reported. Nevertheless, there are only four previous case reports of patients who underwent hepatectomy for HCC due to poorer cardiac function, higher central venous pressure (CVP), and liver cirrhosis caused due to congestion. Herein, we report a case of a 37-year-old female patient with HCC and FALD who was successfully treated by hepatectomy while reducing the CVP.

## Case presentation

**Fig. 1:**
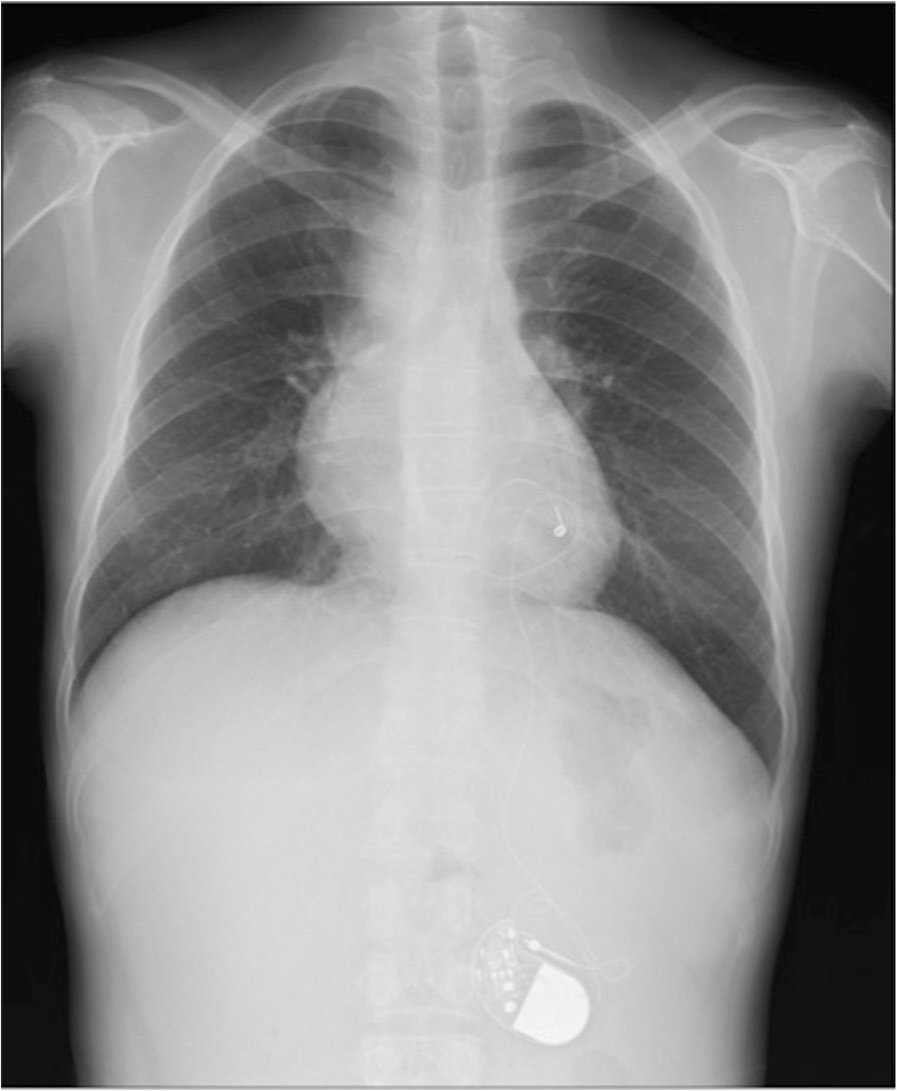
Chest X-ray showed no congestion of bilateral lung field or a globular heart. Pacemaker is located at left upper quadrant

**Fig. 2:**
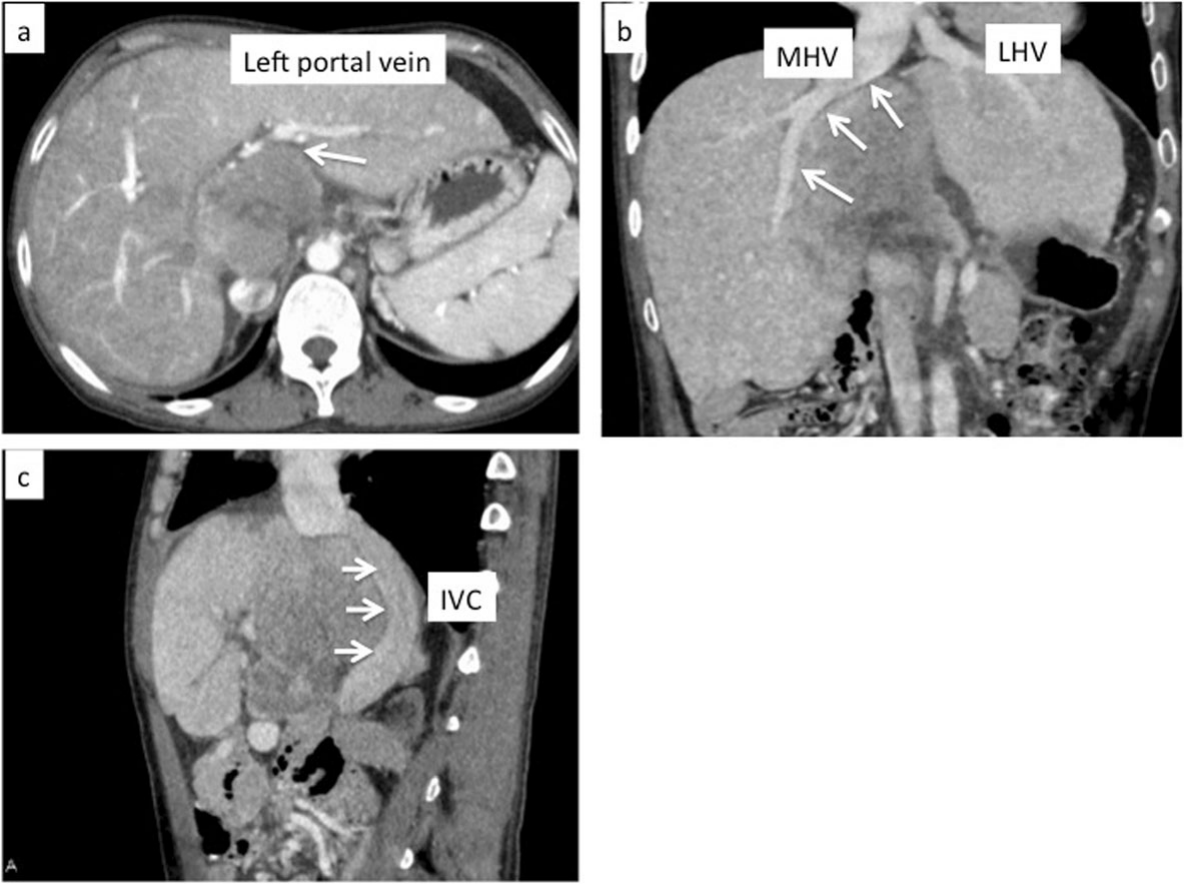
Computed tomography showed a tumor attached to left portal vein (**a**). The tumor also attached to middle hepatic vein (MHV) (**b**) and inferior vena cava (IVC) (**c**)

The patient was born with a complex congenital heart defect with double outlet right ventricle, pulmonary valve atresia, and endocardial cushion defect. At the age of 6 years, she had undergone the Fontan procedure with tricuspid annuloplasty and ligation of patent ductus arteriosus and left superior pulmonary vein ligation at our hospital. At the age of 11 years, she had received a heart pacemaker insertion procedure for complete atrioventricular block. Recently, at age 37 years, she visited a local hospital with a complaint of epigastralgia, wherein a liver tumor was detected. Noninvasive blood pressure was 110/73 mmHg. Her weight was 43 kg, and her height was 157 cm. There was no abdominal distension or tenderness. Chest X-ray revealed a clear lung field and a globular heart, with the pacemaker located in the left upper quadrant (Fig. 1). Computed tomography (CT) revealed a tumor measuring 80 mm in diameter in the caudate lobe. This tumor demonstrated low density on the arterial phase CT scan and low density on the venous phase CT scan. It was located in close proximity to the left portal vein (Fig. 2a) and attached middle hepatic vein (Fig. 2b) and inferior vena cava (IVC) (Fig. 2c). Enlargement of the liver and splenomegaly were also detected, but there was no ascites and collateral vessels. Serum levels of the tumor makers alpha-fetoprotein (AFP) and protein induced by vitamin K antagonists-II (PIVKA-II) were elevated to 81,663 ng/ml (normal < 10 ng/ml) and 238 mAU/ml (normal < 40 mAU/ml), respectively. Other laboratory examinations were within normal limits, except alkaline phosphatase whose level was 132 U/l (normal 6–46 U/l). Tests for both hepatitis B and C and autoimmune markers showed negative results. Indocyanine green retention rate was 6% at 15 min. To perform hepatectomy safely for the patient, cardiac assessment was undertaken by cardiovascular physicians. Chest examination demonstrated no murmurs, rubs, or gallops, except normal S1 and S2 sounds. Oxygen saturation in room air was 87–93% with a respiratory rate of 14 breaths per minute. Cardiac catheter test was performed to assess her accurate heart functions, which revealed a CVP of 12 mmHg, a cardiac output of 2.5 l/min, a left ventricular ejection function of 56%, common atrioventricular valve regurgitation with grade I, and a right atrium volume of 48.3 ml/m^2^. After an extensive multidisciplinary discussion involving experts in adult and congenital cardiology, cardiovascular surgery, and anesthesiology, it was decided to perform hepatectomy for this patient after obtaining the informed consent.

Before the surgery, an arterial line, a central venous catheter, and a Swan-Ganz catheter were inserted and a transesophageal echocardiogram probe was placed.

**Fig. 3:**
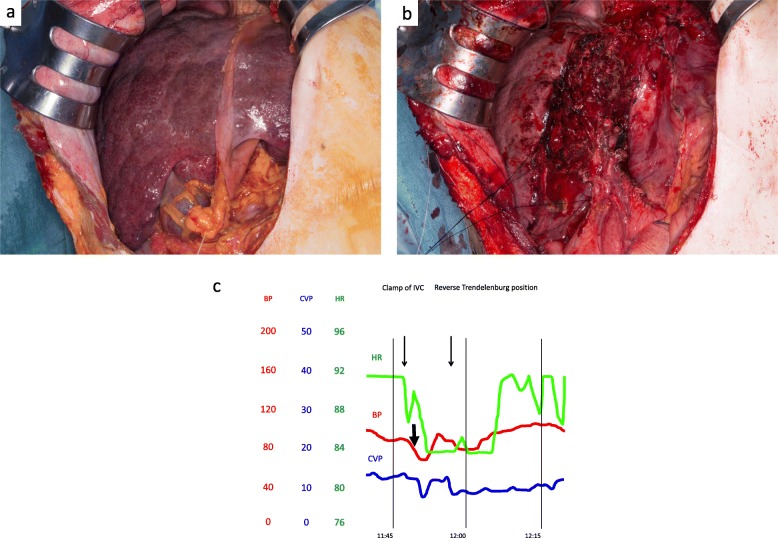
The liver is cirrhosis due to congestion (**a**). After, left hepatectomy and caudate lobectomy were shown (**b**). We tried half-clamping of the inferior vena cava (IVC) below the liver. However, systemic blood pressure decreased below 80 mmHg. Therefore, we avoided IVC clamp and underwent to the reverse Trendelenburg position (rTP). This procedure reduced CVP from 12 to 10 mmHg without a decrease in the systemic blood pressure (**c**)

**Fig. 4:**
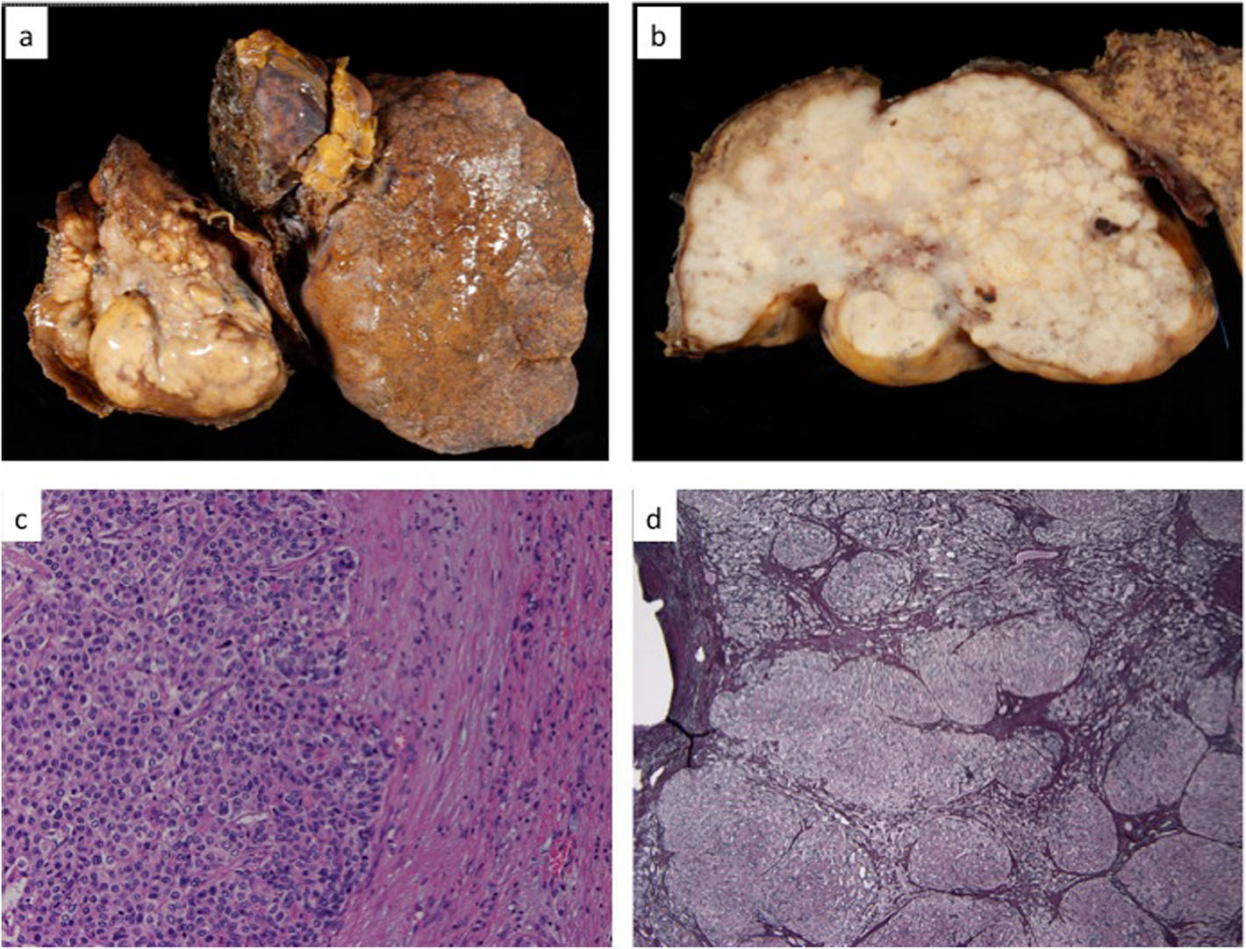
Macroscopically, this tumor showed 80 mm in diameter with white color area with liver cirrhosis (**a**, **b**). Microscopically, tumor cells showed a poorly differentiated HCC (hematoxylin and eosin staining × 10) (**c**). Noncancerous liver tissue was liver cirrhosis due to congestive liver (silver staining)

A J-shaped incision was made by carefully checking the pacemaker leads that were located in the left upper quadrant of the abdominal wall. Surface roughness with congestive liver was observed, and a tumor was confirmed in the caudate lobe (Fig. 3a). Next, we tried half-clamping of the inferior vena cava (IVC) below the liver. However, systemic blood pressure decreased below 80 mmHg, and CVP decreased below 10 mmHg (Fig. 3c). Therefore, we avoided IVC clamp and underwent reverse Trendelenburg position (rTP). This procedure reduced CVP from 12 to 10 mmHg without a decrease in the systemic blood pressure (Fig. 3c). Left hepatectomy and caudate lobectomy were performed without any occurrence of a cardiac event (Fig. 3b). The total operation duration was 450 min, with a total blood loss of 3.2 l. Her hemodynamic status was stable with adequate urine output throughout the operation. Her postoperative course was also uneventful, except ascites that required diuretics, and she was discharged 9 days after the surgery. Macroscopic findings revealed an irregular white tumor (Fig. 4a, b), which was diagnosed as a poorly differentiated HCC (Fig. 4c). Noncancerous liver tissue was liver cirrhosis due to congestion (Fig. 4d). At 16 months after the surgery, the patient is still alive and is undergoing targeted molecular therapy for lung metastasis.

## Discussion

The Fontan procedure has been widely accepted for children with congenital heart defects resulting in single ventricle physiology [3]. Long-term liver dysfunction develops as a late complication after the Fontan procedure, which has been termed as FALD. It is believed that liver fibrosis or cirrhosis caused due to congestion is one of the causes of FALD based on the observation that the systemic venous pressure was 3- to 4-fold higher than normal in patients who have undergone the Fontan procedure [2]. The development of HCC in young patients with FALD has also been recently reported. Till date, there have been 23 past reports of patients with HCC after the Fontan procedure [3–16] (Table 1), wherein the majority of patients were still young, but curative treatments could not be performed due to liver cirrhosis and poor cardiac functional reserve. Six patients were treated by transarterial chemoembolization or transarterial embolization, and another six were treated with systemic chemotherapy. Unfortunately, only one patient was treated with best supportive care, and only five patients underwent hepatectomy for HCC [3, 11–13, 16].

**Table 1:** Case of HCC with FC

Reference	Age/sex	Tumor size (cm)	HBV/HCV	AFP (ng/ml)	LVEF (%)	CVP (mmHg)	Treatment	Operative tips	Outcome
3	24/F	10.8	−/−	50,000	58	14	L hepatectomy	All the inflow and outflow vessels were dissected and slung	Dead, 6 months
4	27/F	22.1	−/−	162.7	ND	ND	5 FU		Dead, 1 year
4	28/F	4	−/−	Negative	ND	ND	Sorafenib		Dead, 1 year Liver failure
5	27/F	2.2	−/−	162.7	ND	ND	Systemic therapy		Dead, 1 year
5	28/F	4	−/−	788	ND	ND	Sorafenib		Dead, 1 year
6	32/F	4	−/−	700	ND	ND	TACE		Alive
6	24/M	ND	−/−	5000	ND	ND	ND		Dead
6	33/M	ND	−/−	630	ND	ND	Radioembolization		Dead
6	42/F	ND	−/+	106	ND	ND	TACE		Alive
7	51/M	1	−/−	WNL	ND	ND	Local abration		Alive, 28 months
8	19/F	ND	−/−	ND	ND	ND	Sorafenib		Dead, 3 months
9	15/M	ND	−/−	2	ND	ND	TAE		Dead, 2 years
10	16/F	12.5	−/−	211,580	59	ND	Systemic chemotherapy		Dead, 2 months
11	23/F	14.8	ND	ND	ND	ND	Extended R-H	Venovenous bypass	ND
12	32/M	4	−/−	13	ND	12–13	S6 and S7 resection	Pringle maneuver	ND
13	29/F	1.5	−/−	117.1	ND	ND	S4 wedge resection	Not mentioned	Alive, 1 year
14	24	ND	−/−	ND	ND	17	ND		ND
14	41	ND	−/+	ND	ND	ND	TAE		ND
14	22	ND	−/−	ND	ND	19	TAE + systemic chemotherapy		ND
14	29	ND	−/−	ND	ND	21	No indication		Dead
14	43	ND	−/−	ND	ND	13	TAE + systemic chemotherapy		Dead
15	13/F	5	−/−	3340	ND	ND	TAE with lipiodol and doxorubicin eluting microbeads		Alive, 6 months
Present case	37/F	6.3	−/−	81,663	56	12	L hepatectomy with caudate lobectomy	rTP and Pringle maneuver	Alive, 16 months with lung metastasis

*AFP* Alpha-fetoprotein, *ND* not described, *TACE* transarterial chemoembolization, *TAE* transarterial embolization, *WNL* within normal limits, *S* segment, *R* right, *L* left

For performing safe hepatectomy, the Pringle maneuver and reduction of CVP are considered as useful methods for controlling operative blood loss. For performing hepatectomy in patients with FALD, reduction of CVP is important because a high CVP is a risk factor for uncontrollable bleeding during hepatectomy. Weyker et al. reported that an extended right hepatectomy with venovenous bypass could be performed to reduce blood loss, which resulted in a blood loss of 1000 ml [11]. Lo et al. reported that ligation of inflow and outflow vessels before performing left hepatectomy resulted in a blood loss of 4100 ml [3]. For the patient described in this case report, we performed left hepatectomy and caudate lobe resection in the reverse Trendelenburg position under low CVP anesthesia. We observed that although CVP reduced from 12 to 10 mmHg and there was good control of bleeding from the hepatic veins in the cut surface and around the IVC, the total blood loss was 3200 ml.

The procedure of IVC clamping below the liver is known to reduce CVP and blood loss from the cut surface during hepatectomy. IVC clamping may be useful because patients with FALD generally have a high mean CVP. However, this procedure often results in a reduction of blood pressure. Yoneda et al. reported that the reverse Trendelenburg position is a safer technique for lowering CVP without decreasing the blood pressure than IVC clamping [17]. Therefore, we preferred this position over IVC clamping as the latter procedure is known to decrease blood pressure.

Liver dysfunction occurring after the Fontan procedure is known as FALD. Hepatic fibrosis or cirrhosis caused due to congestion is the primary cause of liver dysfunction. According to a Japanese nationwide survey, the mortality rate from liver diseases in these patients was only 0.19%; however, if the patients had LC and/or HCC, the mortality rate increased to approximately 30%. The researchers of that survey concluded that LC and/or HCC in patients undergoing the Fontan procedure were not rare late complications and were associated with high mortality rates [14]. Egbe et al. also reported that FALD-associated HCC has poor prognosis compared with other etiology. They also mentioned that HCC could occur rapidly in the Fontan population [18].

## Conclusions

First left hepatectomy with caudate lobectomy during reverse Trendelenburg position which reduced the CVP was performed in a patient with HCC and FALD.

## Data Availability

The authors declare that all the data in this article are available within the article.

## Abbreviations

CVP: Central venous pressure
FC: Fontan circulation
AV: Atrioventricular
rTP: Reverse Trendelenburg position
cIVC: Inferior vena cava clamp
AFP: Alpha-fetoprotein
TACE: Transarterial chemoembolization
TAE: Transarterial embolization
